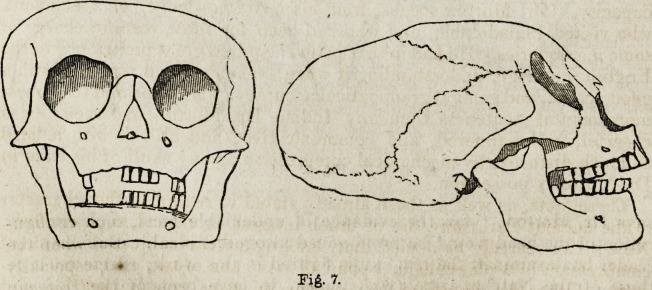# Crania Americana; or a Comparative View of the Skulls of Various Aboriginal Nations of North and South America; with an Essay on the Varieties of the Human Species

**Published:** 1840-10

**Authors:** 


### ART. IX.

*Crania Americana; or a Comparative View of the Skulls of various Aboriginal Nations of North and South America; to which is prefixed an Essay on the Varieties of the Human Species.* By SAMUEL GEORGE MORTON, M.D. Professor of Anatomy in Pennsylvania College, &c.—*Philadelphia*, 1839. Folio, pp. 296. *With Seventy-eight Lithographed Plates, and a Coloured Map.*

WE have often regretted that the opportunities of original research which so abound in a newly-settled country have been so scantily embraced by our medical brethren of the United States; and that, in their zeal for the acquirement of the treasures of foreign science, they should so far overlook those in their own possession, of which they may justly be called upon to render an account. The work before us, however, goes far to redeem the national character; for it is one which any country might be proud to have produced. It consists of a series of delineations of the most characteristic specimens, taken from a much larger number in Dr. Morton's possession or placed by friends at his command, of the various aboriginal nations of the American continent; several of which are now extinct (some having disappeared many centuries since), and others are rapidly becoming so. These delineations have evidently been executed with great care. The fidelity of the outlines was secured by a mechanical contrivance; and so much attention has been paid by the author to their accurate completion, that many of the plates have been drawn the second and even the third time; and in several instances the entire edition was cancelled in order to correct inaccuracies that had previously escaped observation. As specimens of art they may rank very high; and having had occasion to speak more than once in rather depreciating language of American wood-engraving, we gladly take the opportunity of giving this example of American lithography its due meed of praise.

The volume is dedicated to our illustrious countryman Dr. Prichard, "the learned and ingenious author of 'Researches into the Physical History of Mankind.'" We need scarcely add our own opinion that to no one could this work, "which is designed to illustrate a portion of the same interesting enquiry," be more appropriately inscribed. A second dedication is added, to the author's uncle, Mr. James Morton, of Clonmell, Ireland, by whose liberal assistance, in conjunction with that of another friend, he was able to extend his work far beyond his first intention.* "I claim, however, some merit," says Dr. Morton, with a justifiable pride, "for having commenced publication when my subscription list bore but fifteen names; and I persisted for a long time on my own resources, although frequently apprehensive that an enterprise which never had gain for its object would add pecuniary loss to numberless vexations." We rejoice to be able to adduce such an instance of disinterestedness on the part of a member of our profession, in a country where, according to the testimony of some veracious (?) travellers, no conversation is ever carried on between gentlemen for a quarter of an hour in which the word *dollar* is not often repeated. But, judging from his second dedication, Dr. Morton's connexion with the "old country" would not seem very remote, and we would fain regard his work as of British origin.

To offer an analysis of such a work without those illustrations in which its chief interest lies would be unsatisfactory to ourselves and unprofitable to our readers. But our desire to present them with some specimens of its character has led us to select a few of the more interesting statements, which will be rendered intelligible by the subjoined outlines. We regard the great excellence of the work as consisting in the *data* with which it furnishes the anthropologist. Dr. Morton's own inferences from these must be carefully separated from them; and we would not be understood as always according with the opinions which we transfer from his pages. He appears to us somewhat deficient in the palæological knowledge which is necessary for the accurate interpretation of the facts he has collected; but we prefer not at present entering upon the many ethnographical questions which he starts, since we feel assured that they will be discussed with all the aid that profound learning and unwearied industry can give, in the forthcoming volume on the American Nations, in Dr. Prichard's Physical History of Man.

As, for the reason just stated, our object is not to give a particular account of the whole volume, but to select from it some of its most interesting novelties, we may pass over with slight notice the introductory essay on the varieties of the human species, since it contains little that will be new to the readers of Dr. Prichard's elaborate treatise. The author does not grapple with the question of the *unity* or *multiplicity* of the species of man; but contents himself with expressing his belief that the different *races* were "adapted from the beginning to their respective local destinations," and that "the physical characteristics which distinguish them are independent of external causes." He forms twenty-two families or "groups of nations, possessing to a greater or less extent similarity of physical and moral character and language;" and these families

* We learn, however, from a review of this work, in the American Journal of Science, that these dedications are only inserted in those copies which were destined for foreign circulation; and that in the American edition they are replaced by others, to Dr. Ruschenberger and Mr. J. S. Phillips, from whom the author received much assistance in his laborious researches. This strikes us as a very ingenious way of paying four compliments at once, and reminds us of some Edinburgh theses we have seen, in which the dedications occupied nearly as many leaves as the composition to which they were prefixed.

he classifies under the five races indicated by Blumenbach. The following is his general view of the American race.

"It appears to me that the most natural division of the American race is into two families; one of which, the Toltecan family, bears evidence of centuries of demi-civilization; while the other, under the collective title of the American family, embraces all the barbarous nations of the New World, excepting the Polar tribes or Mongol Americans. Some writers, however, suppose even the Esquimaux to be a part of the same original stock, partly because there is some resemblance in features, partly from partial analogy of language, and partly again from a determination to merge the American in the Mongolian. It is obvious, nevertheless, that the continent of America was originally peopled, as it yet is, by a very distinct race; and that the Esquimaux arriving in small and straggling parties from Asia, necessarily adopted more or less of the language and customs of the people among whom they settled: hence the Esquimaux, and especially the Greenlanders, are to be regarded as a partially mixed race among whom the physical character of the Mongolian predominates, while their language presents obvious analogies to that of the Chippewyans who border them to the south." (p. 63.)

We shall not follow our author in his description of the characters of the American family, which includes all the aboriginal tribes of North America, with the exception of the Esquimaux; but shall content ourselves with quoting the following fact with which he concludes it, this being of much psychological interest.

"One of the most remarkable intellectual defects of the Indians is a great difficulty in comprehending anything that belongs to numerical relations. Humboldt states that he never saw a man who might not be made to say that he was eighteen or sixty years of age. Wafer made the same remark in reference to the Indians of Darien; and Mr. Schoolcraft, the United States' Indian agent, assures me that this deficiency is a cause of most of the misunderstandings in respect to treaties entered into by our government and the native tribes. The latter sell their lands for a sum of money without having any conception of the amount; so that if it be a thousand dollars or a million, few of them comprehend the difference until the treaty is signed and the money comes to be divided. Each man is then for the first time made acquainted with his own interest in the transaction, and disappointment and murmurs invariably ensue." (p. 83.)

The history of the Toltecan family, which is now extinct as a separate group, offers many points of peculiar interest to the anthropologist. At the time of the Spanish invasion, according to Dr. Morton, it occupied Mexico, which may perhaps be regarded as its centre, the southern part of North America, and the northern and eastern coasts of South America. Between this and the American family there was obviously a great difference in regard to intellectual powers. In the arts and sciences of the Toltecans we have evidences of an advanced civilization. From California to the southern extremity of Peru their architectural remains are everywhere encountered, to surprise the traveller and confound the antiquary: among these are pyramids, temples, grottos, bas-reliefs, and arabesques; whilst their roads, aqueducts, and fortifications, and the sites of their mining operations sufficiently attest their knowledge of the practical arts of life.

"In assigning the geographical limits of the Toltecan family it is not to be supposed that they alone inhabited this extended region; for while successive nations of that family held dominion over it for thousands of years other and barbarous tribes were everywhere dispersed through the country; and, whether of aboriginal or exotic origin, may have at all times constituted a large part of the population. During these periods of power and greatness an organized feudal system divided the nation into two great classes of nobles and plebeians; and there appears to have been as much objection to the amalgamation of these classes as ever existed in an aristocratic state of Europe. The advent of the Spaniards destroyed all distinctions by reducing both classes to equal vassalage; and three centuries of slavery and oppression on the part of the Spaniards have left few traces of Mexican and Peruvian civilization, excepting what we glean from their history and antiquities. These nations can no longer be identified in existing communities; and the mixed and motley people who now bear those names are as unlike their ancestors in moral and intellectual character as the degraded Copts of Egypt are unlike their progenitors of the age of Pharaoh." (p. 84.)

The mixture of races in America, resulting from the introduction of Europeans and negroes as integral parts of its population, is well known. There are two remarkable tribes, however, mentioned by Dr. Morton, which are restricted to particular localities and with which we are less familiar. Of these, one is that known by the name of the *Mamelukes* of San Paulo, in Paraguay. They are the offspring of Indian women by men of various nations of Europe. The fathers were often outlaws; the mothers the very refuse of the Indian tribes. It is not surprising, therefore, that a race springing from these highly respectable progenitors should be distinguished for its barbarity; and the tribe was for a long time the terror both of European settlements and of the native races. Extensive districts were depopulated, some of the inhabitants being slain and others reduced to slavery; and these atrocious practices were only done away at last by the severest measures on the part of the parent governments of Spain and Portugal. Allied in origin to these are the *Confusos* of Brazil, a numerous community with long and curled hair, "a mean between the wool of the negro and the long stiff hair of the American," from which their origin is sufficiently evident.

The account of the individual crania commences with those brought from Peruvian sepulchres. The great number of dead bodies remaining in a desiccated state in the arid parts of this district has been a subject of surprise to all travellers, and serves to convey an idea of the vast population that has at different times derived its subsistence from that country. We are told, for example, by Wafer, an intelligent voyager, that having landed at Vermejo, in Peru, in the year 1687, he found the vicinity of the town so strewed with desiccated bodies that, in his own language, a man might have walked a mile and a half and trod on them at every step. The opportunities of obtaining crania are, therefore, sufficiently abundant, and Dr. Morton has examined nearly a hundred. From this examination he arrives at the interesting conclusion, which harmonizes with that handed down by tradition, "that Peru appears to have been at different times peopled by two nations of differently formed crania, one of which is perhaps extinct, or at least exists only as blended by adventitious circumstances in various remote and scattered tribes of the present Indian race. Of these two families, that which was antecedent to the appearance of the Incas is designated as the *ancient Peruvian*, of which the remains have hitherto been found only in Peru, and especially in that division of it now called Bolivia."

The skulls of these ancient Peruvians are small, very much elongated, narrow along their whole length, with a very retreating forehead; the face projects, and the upper jaw is thrust forwards. The flattening of the forehead is so great as to suggest the idea of artificial compression. In some instances, however, no distinct traces of it can be remarked. Dr. Morton justly observes that "when the forehead of a naturally rounded head has been much compressed by art, the back and lateral parts of the cranium become proportionally expanded in order to make room for the brain that has been displaced from the anterior chamber." This may be observed in every cranium that bears unequivocal marks of artificial compression. Now the heads of these ancient Peruvians seldom present such lateral expansion, but on the contrary are as remarkable for their narrowness as their length. Of their general form the outline (fig. 1) will convey an idea. But artificial compression was undoubtedly practised in some instances; as in the skull of which fig. 2 gives the outline. On this Dr. Morton remarks,

Fig. 1.

Fig. 2.

"It is a feature both of civilized and savage communities to admire their own national characteristics above all others; and hence, where nature has denied an imaginary grace art is called in to supply the deficiency, and even where there has been no such deficiency, human vanity prompts to extravagance. Thus I have seen skulls of this race which must have been naturally very low and long, yet in order to exaggerate a feature that was considered beautiful, compression has been applied until the whole head has assumed more the character of the monkey than the man." (p. 100.)

From the opinions expressed in the volume before us, we gather that Dr. Morton was inclined to regard the skull here outlined as fig. 1, as a type of the cranial conformation of the ancient Peruvian race; and he accordingly remarks,—"It would have been natural to suppose that a people with heads so small and badly formed would occupy the lowest place in the scale of human intelligence: such, however, was not the case;"—for there is distinct evidence that civilization existed in Peru anterior to the advent of the Incas, which may be dated at about the year 1100; and that many of the monumental remains which are most remarkable for their architecture and sculpture may be traced to the very nation whose extraordinary crania we have been considering. Against this nation the Incas appear to have waged a war of extermination; but some remains of it seem to have existed at the time of the Spanish invasion. But we learn from the Review already alluded to that Dr. Morton has since changed his opinion; and that, although still satisfied that fig. 1 represents an unaltered cranium, he now believes it to be the *only unaltered* one he has found, and therefore not sufficient for the establishment of the national type. "My matured opinion is," he says, "that the ancient Peruvians were a branch of the great Toltecan family; and that the cranium had the same general characteristics in both. I am at a loss to conjecture how they *narrowed the face* in such due proportion to the head; but the fact seems indisputable."

What was the national character of the *unaltered* cranium of the ancient Peruvians, must still therefore remain a matter of doubt, and no inferences can fairly be drawn from the *single* skull figured by Dr. M. in regard to the want of correspondence between cerebral development and national character, as deduced from traditional and monumental remains. To the question how far the character would be influenced by artificial compression, we shall presently advert.

Dr. Morton thinks that there is good reason to believe that the Incas were a part of the Toltecan nation, which was the most civilized tribe of ancient Mexico; and which, after governing it for four centuries, suddenly abandoned it about the year 1050, in consequence of a series of national calamities which gave a fatal blow to their prosperity and power. The Toltecans migrated in large bodies to various parts of the continent; and the coincidence in point of time would at once lead to the idea that the Incas were a part of the tribe. This is borne out by the correspondence in national character and in the form of their crania. Of all the nations of the new world the Toltecans had attained the highest degree of civilization. They lived in society, collecting themselves into cities, under the government of kings and regular laws. They were not remarkably warlike, and preferred the cultivation of arts to the exercise of arms; they also devoted themselves to architecture, and cultivated with care various useful plants and fruits. Nor did they practise those arts only which are considered as necessary to human comfort, for they attended also to those which minister to luxury. The following is Dr. Morton's account of their crania.

"The skull of these people is remarkable for its small size, and also for its quadrangular form. The occiput is greatly compressed, sometimes absolutely vertical; the sides are swelled out; and the forehead is somewhat elevated, but very retreating. The capacity of the cavity of the cranium, derived from the measurement of the many specimens of the pure Inca race, shows, as we shall hereafter see, a singularly small cerebral mass for an intelligent and civilized people. Their heads are remarkable, not only for their smallness but for their irregularity; for in the whole series in my possession there is but one that can be called symmetrical. This irregularity chiefly consists in the greater projection of the occiput to one side than the other, showing in some instances a surprising degree of deformity. As this condition is as often observed on one side as on the other, it is not to be attributed to the intentional application of mechanical force; and, on the contrary, it is to a certain degree common to the whole American race, and is sometimes no doubt increased by the manner in which the child is placed in the cradle." (p. 115.)

In fig. 3 is shown the outline of a well-characterized Peruvian head; and in fig. 4 is represented the extraordinary deformity in the occipital region of the head of a child. This kind of deformity may be noticed

Fig. 3.

Fig. 4.

at the present day in the heads of many South Sea Islanders; and is there obviously occasioned by the very extraordinary position in which the child is carried by its nurse, which causes a constant pressure on one side of the occiput. The system of artificial flattening appears to have continued among the lower orders of Peruvians, however, after their subjugation by the Incas. This was effected, by means of boards and ligatures, in two ways: Either the forehead and occiput were compressed, so that the head became broad from side to side (which, perhaps, may have been with the view of conforming it to the aristocratic standard); or the compression was applied in such a manner as to elongate the head from back to front, which was thought to give an additional ferocity to the countenance, desirable for warlike purposes. The Ecclesiastical Court of Lima passed a decree in the year 1585, forbidding parents, under certain specified penalties, to compress or distort the heads of their children in the various modes which were then in vogue. But the power of the church was not effectual in checking the practice, for it exists among some tribes of Indians at the present day.

The Cyclopean structures erected in Peru during the government of the Incas will bear comparison with those of ancient Egypt; and the wonder is increased when it is recollected that no beast of burthen but the Llama existed in Peru before the Spanish invasion. "At a time when a public highway was either a relic of Roman greatness or a sort of nonentity in England, there were roads of 1500 miles in length in the empire of Peru, carried over heights which overtop the peak of Teneriffe. The feudal system was as firmly established in these transatlantic kingdoms as in France. The Peruvians were ignorant of the mode of forming an arch; but they had constructed suspension bridges over frightful ravines: they had no implements of iron, but their forefathers could move blocks of stone as huge as the sphinxes and Memnons of Egypt." Among the evidences of their civilization, it may be mentioned that, in consequence of the frequency of infanticide, foundling-hospitals were established by the government, in which children were received and provided for at the public expense. The subjugation of such a powerful people by a "handful of brigands," as Dr. Morton terms the invading troop of Pizarro (consisting of sixty-two horsemen and 102 foot-soldiers, of whom twenty were armed with cross-bows and three with muskets), is one of the most remarkable events in the history of nations. It was probably only a repetition, however, of the scene that had been acted four centuries previously, when the ancient Peruvians were invaded by the Toltecan emigrants.

Of the physiognomy of the Toltecans, we have some curious remains in bas-reliefs executed by them during their sojourn in Mexico. From one of these the subjoined figures (fig. 5) are copied. "Were it not,"

Fig. 5.

says Dr. Morton, "for the evidence of undeniable facts, such configuration of the head would be pronounced altogether ideal. But when the reader has examined the real skulls figured in this work, and especially those of the Natchez tribe (who appear to have been of the Toltecan stock), he will perceive in them a distortion similar in kind to that represented in the bas-reliefs of Palenque, but in a much more exaggerated degree." Outlines of the profile and front views of one of these skulls are given in fig. 6. That the tribe of Natchez Indians was a branch of the Toltecan family, appeared from their traditions and usages. Their

Fig. 6.

migration took place northwards from Mexico; and they allocated themselves at the south-western corner of North America, principally in Florida. They were exterminated by the French in the year 1730. The singular form of their heads was partly due to artificial compression; employed, probably, under the idea of increasing a natural grace. This was effected by means of a bag of sand placed upon the forehead, whilst the occiput lay upon a sort of mould, of which it gradually took the form under the slow but constant influence of this pressure.

In contrast with the vertical flattening of these crania, we may notice the extraordinary horizontal flattening practised by the tribes bordering on the Columbia river, termed "Flat-heads." The cranium, of which a lateral and anterior view is given in fig. 7, is the most extraordinary ex-

Fig. 7.

ample of this compressing process which has come under Dr. Morton's observation. The vertical diameter is reduced to little more than four inches; the top of the cranium presents a flattened arch not far removed from a horizontal plane; and the face is protruded until the facial angle is reduced to 66°, the lowest grade probably ever observed in a human skull. The compression has also destroyed, in a remarkable degree, the lateral symmetry of the skull. The following account of the mode in which it is effected, is quoted by Dr. Morton from the account given by Mr. Townsend, who journeyed among these tribes:

"The mode in which the flattening is effected varies considerably with the different tribes. The Wallamet Indians place the infant soon after birth upon a board, to the edges of which are attached little loops of hempen cord or leather, and other similar cords passed across and back, in a zigzag manner, through these loops, inclosing the child, and binding it firmly down. To the upper edge of this board, in which there is a depression to receive the back part of the head, another smaller one is attached by hinges of leather, and made to lie obliquely upon the forehead; the force of the pressure being regulated by several strings attached to its edge, which are passed through holes in the boards upon which the infant is lying, and secured there.

"'The modes of the Chinouks and others near the sea differs widely from that of the upper Indians, and appears somewhat less barbarous and cruel. A sort of cradle is formed by excavating a pine-log to the depth of eight or ten inches. The child is placed in it on a bed of little grass mats, and bound down in the manner above described. A little boss of tightly plaited and woven grass is then applied to the forehead and secured by a cord to the loops at each side. The infant is thus suffered to remain from four to eight months, or until the sutures of the skull have in some measure united, and the bone become solid and firm. It is seldom or never taken from the cradle, except in case of severe illness, until the flattening process is completed.'

"Either of the preceding processes," continues Dr. Morton, "must be very painful, often giving rise to ulceration of the scalp, and perhaps not unfrequently to death itself; yet so highly is this deformity valued among the Columbia river tribes, that their slaves (who are for the most part derived from the adjacent tribes) are not allowed to practise it. The appearance of the infant during the process is described as both ludicrous and frightful; 'its little black eyes, forced out by the tightness of the bandages, resemble those of a mouse choked in a trap.'" (p. 205.)

According to the concurrent testimony of all travellers who have visited them, these "flat-head" tribes are remarkable for their acuteness and capacity. Dr. Morton speaks, from his own knowledge, of a Chenouk who visited Philadelphia, and who had been for three years in charge of some missionaries. He had in that period acquired great proficiency in the English language, understanding it when spoken to, and replying with a good accent, and general grammatical accuracy. He appeared to possess more mental acuteness than any Indian Dr. Morton had seen; was cheerful, well mannered, and communicative; and all this with a head as much distorted by mechanical compression as any skull of his tribe in Dr. Morton's possession.

These facts, as well as those already stated in regard to the character of the ancient Peruvians, are of much interest in regard to phrenology. Now it can scarcely be denied that, looking at the subject of artificial compression in a phrenological point of view, one of two effects must result: Either the growth of some organs must be greatly impeded and that of others encouraged, in which case the influence on the character ought to develope itself as the individual approaches adult age; or the relative position of the organs will be so changed that common rules for admeasurement will not apply to them; and then the question arises whether these rules are applicable to uncompressed crania (such as that in fig. 1), of which the form so much resembles that of the skulls that have been submitted to the process. If not, they need to be greatly modified before their results can be *certainly* depended on in *any* case, even within wide limits.

It will be remarked, however, that these observations do not apply to those fundamental principles with which we have, on a former occasion, expressed our concurrence; but only to the independent question, which we must regard as still *sub judice*, of the degree of certainty with which the relative size of the several organs can be predicated from the external configuration of the cranium. The question may be put in this simple form: "Here are two similar crania (e. g. ancient Peruvian), whose aspect indicates a very low degree of intellectual development. There is extraneous reason, however, for the belief that the form of one of these is natural and of the other artificial. How can their respective mental capabilities be predicated, and what difference would the phrenologist make in his estimate of their characters, if (as in many of Dr. Morton's crania) there are no decided indications that either is abnormal?" This is a question to be decided by further observations; and in the mean time we may quote the following from the American Journal of Science:

"Mr. George Combe mentioned in his late lectures at Newhaven, that he had examined the head of a young Indian of about twenty years of age, from the Columbia river. He found the parietal diameter actually greater than the frontal and occipital; the cranium having been compressed, by means of a board and cushions, in infancy. The organs in the superciliary ridge were fully developed; the upper part of his forehead was flat and deficient; his organs of language and form were large. He had studied the English language for two years and spoke it tolerably well. Mr. Combe added that in conversation he was intelligent, ready, and fluent on all subjects that fell within the scope of the faculties of observation, situated in the superciliary ridge; but dull, unintelligent, and destitute equally of ideas and language, on topics that implied the activity of the reflecting faculties, situated in the upper part of the forehead. Mr. Combe considered his mental powers to be in direct harmony with the development of his brain."

In this individual case, then, there was a correspondence between the character and the *artificial* form, such as would show that the *relative development* of the several organs of the brain is modified by compression. Whether this always holds good, we are not yet in a condition to determine. The very decided testimony of travellers to the intellectual powers of these tribes, would seem to render it probable that *displacement* must also occur; in which case there would be a want of that harmony *here* noticed between the external form of the cranium and the capacity of the individual.

At the conclusion of the volume we find a very elaborate series of measurements of the different crania figured in it; on which the phrenological student would do well to bestow much attention, although they do not present any very decided results. Dr. Morton's general opinion, as to the value of the science of phrenology, may be best presented in his own words. The following is an extract from his letter to Mr. Phillips (at the close of our copy, but standing as the epistle dedicatory in the *native* edition), by whom the above measurements were made with great ingenuity and patience:

"In the study of phrenology I am yet a learner; and it appeared to me a wiser plan to present the facts unbiassed by theory, and let the reader draw his own conclusions. You and I have long admitted the fundamental principles of the science, viz: That the brain is the organ of the mind, and that its different parts perform their different functions; but we have been slow to acknowledge the details of cranioscopy as taught by Dr. Gall, and supported and extended by subsequent observers. We have not, however, neglected this branch of the enquiry, but have endeavoured to examine it in connexion with numerous facts, which can only be fully appreciated when they come to be compared with similar measurements derived from the other races of men. Yet I am free to acknowledge that there is a singular harmony between the mental character of the Indian and his cranial development, as explained by phrenology."

Appended to Dr. Morton's ethnographical account of the crania is an Essay by Mr. George Combe "on the Relation between the Natural Talents and Dispositions of Nations, and the Development of their Brains." We had hoped to have found in this Mr. Combe's views on several of the points which Dr. Morton regarded as difficulties in the phrenological interpretation of the data adduced by him. But, as we learn from the preface, Mr. Combe furnished the essay under circumstances which precluded his acquainting himself with what Dr. Morton had written; and his knowledge of details was limited to an inspection of the plates, many of which he compared with the original skulls. He has therefore preferred confining himself to general observations, with the view of enabling his readers to draw their own conclusions from Dr. Morton's delineations and measurements; and being limited in space, he has not introduced any views of the subject which will not be found in most elementary treatises on phrenology. This we cannot but regret, as we suspect the phrenological student needs more guidance than he will find here, in order that his conclusions may be satisfactory. The omission of all notice of the question of artificial compression, which we should have thought the sight of Dr. Morton's plates could not but have suggested, is that which chiefly surprises us. With the spirit of the following remarks, however, we fully coincide:

"The harmony or discord between Dr. Morton's historical delineations and the phrenological inductions which the reader will be enabled to draw by applying the rules now to be laid down, will depend upon the degree of approximation of each to nature. Where discrepancies shall appear, one or other of our views must be erroneous. I solicit the reader candidly to investigate both representations, and not to condemn phrenology at once as chargeable exclusively with error. Imperfect historical descriptions have been given of distant nations, and particularly of barbarous and savage tribes, whose manners have been imperfectly observed, and whose language has been scarcely at all comprehended; and it may ultimately be discovered that the characteristics indicated by the size and forms of their brains have been more correct than the hasty impressions of travellers." (p. 270.)

In conclusion, we have only to express again the gratification we have felt in the examination of Dr. Morton's unique and splendid work, and the hope that he will not relax in the prosecution of his interesting and important enquiries. We look forwards with much satisfaction to the continuation which he has given us reason to expect; and hope that he may obtain sufficient encouragement to enable him to complete his account of the Crania Americana in a manner which shall leave little for the anthropologist to desire.

## Figures and Tables

**Fig. 1. f1:**
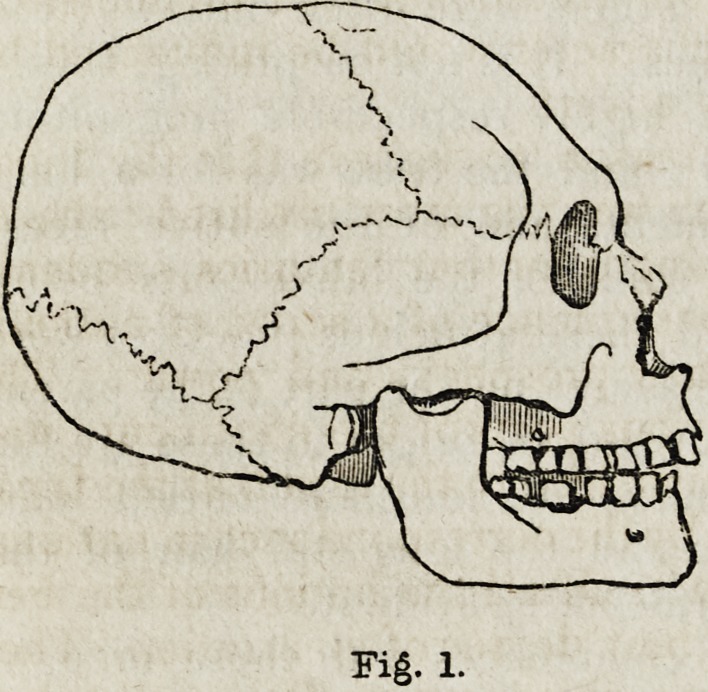


**Fig. 2. f2:**
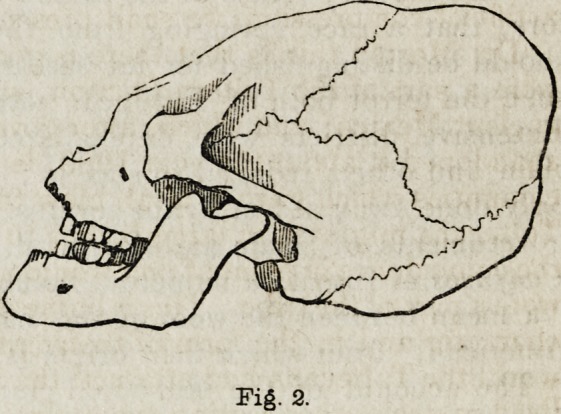


**Fig. 3. f3:**
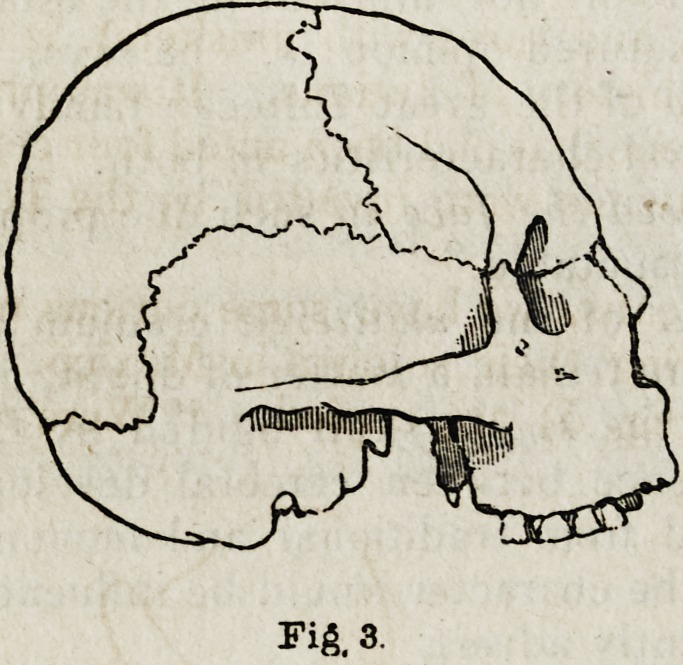


**Fig. 4. f4:**
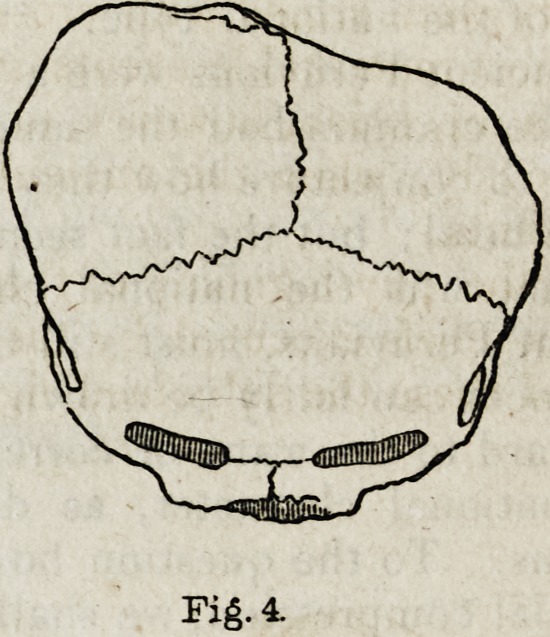


**Fig. 5. f5:**
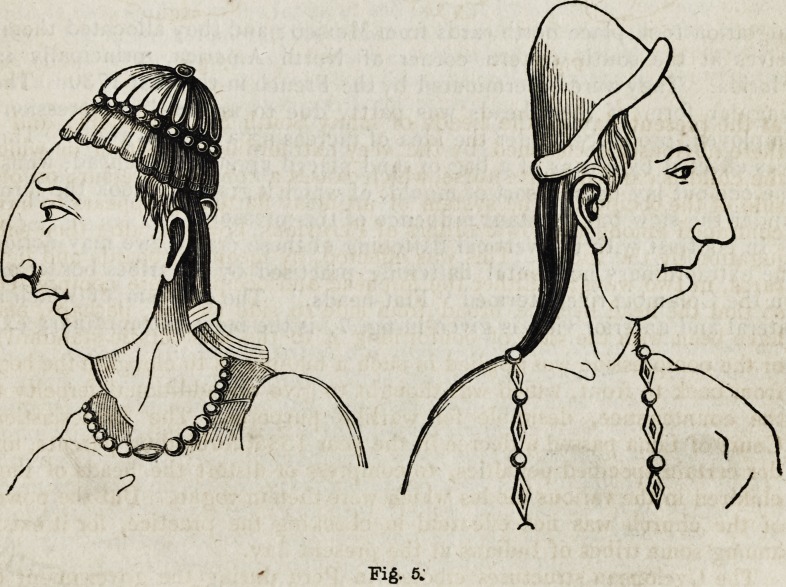


**Fig. 6. f6:**
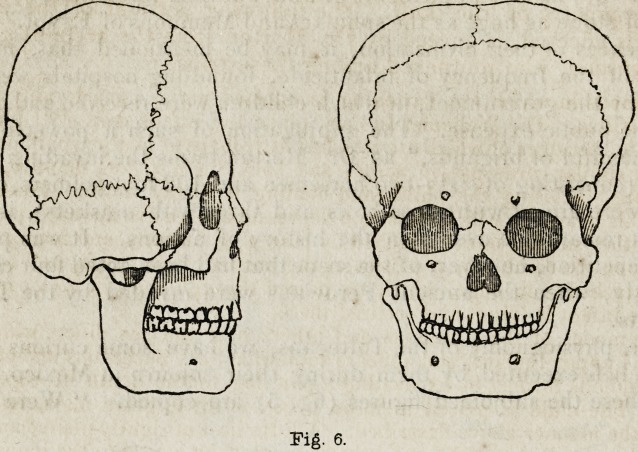


**Fig. 7. f7:**